# Human immunodeficiency virus type 1 viral protein R (Vpr) induces CCL5 expression in astrocytes via PI3K and MAPK signaling pathways

**DOI:** 10.1186/1742-2094-10-136

**Published:** 2013-11-13

**Authors:** Mohitkumar R Gangwani, Richard J Noel, Ankit Shah, Vanessa Rivera-Amill, Anil Kumar

**Affiliations:** 1 Division of Pharmacology and Toxicology, School of Pharmacy, University of Missouri, Kansas City, MO 64108, USA; 2 Departments of Biochemistry, Ponce School of Medicine and Health Sciences, Ponce, PR 00716, USA; 3 Departments of Microbiology, Ponce School of Medicine and Health Sciences, Ponce, PR 00716, USA

**Keywords:** HIV-1, Vpr, CCL5, NF-kB, AP-1, MAPK, PI3K/Akt

## Abstract

**Background:**

Neurocognitive impairments remain prevalent in HIV-1 infected individuals despite current antiretroviral therapies. It is increasingly becoming evident that astrocytes play a critical role in HIV-1 neuropathogenesis through the production of proinflammatory cytokines/chemokines. HIV-1 viral protein R (Vpr) plays an important role in neuronal dysfunction; however, its role in neuroinflammation is not well characterized. The major objective of this study was to determine the effect of Vpr in induction of proinflammatory chemokine CCL5 in astrocytes and to define the underlying mechanism(s).

**Methods:**

SVGA astrocytes were either mock transfected or were transfected with a plasmid encoding HIV-1 Vpr, and the cells were harvested at different time intervals. The mRNA level of CCL5 expression was quantified using real-time RT-PCR, and cell culture supernatants were assayed for CCL5 protein concentration. Immunocytochemistry was performed on HIV-1 Vpr transfected astrocytes to check CCL5 expression. Various signaling mechanisms such as p38 MAPK, PI3K/Akt, NF-κB and AP-1 were explored using specific chemical inhibitors and siRNAs.

**Results:**

HIV-1 Vpr transfected astrocytes exhibited time-dependent induction of CCL5 as compared to mock-transfected astrocytes at both the mRNA and protein level. Immunostained images of astrocytes transfected with HIV-1 Vpr also showed much higher accumulation of CCL5 in comparison to untransfected and mock-transfected astrocytes. Pre-treatment with NF-κB (SC514) and PI3K/Akt (LY294002) inhibitor partially abrogated CCL5 mRNA and protein expression levels as opposed to untreated controls after HIV-1 Vpr transfection. Specific siRNAs against p50 and p65 subunits of NF-κB, p38δ MAPK, Akt-2 and Akt-3, and AP-1 transcription factor substantially inhibited the production of CCL5 in HIV-1 Vpr transfected astrocytes.

**Conclusion:**

These results demonstrate the ability of HIV-1 Vpr to induce CCL5 in astrocytes in a time-dependent manner. Furthermore, this effect was observed to be mediated by transcription factors NF-κB and AP-1 and involved the p38-MAPK and PI3K/Akt pathway.

## Introduction

HIV-1 enters the central nervous system (CNS) very early in the course of the disease and causes productive infection in the perivascular macrophages and microglia of the brain [1-3]. HIV-associated neurocognitive disorders or HAND is a common complication of nervous system with HIV-1 infection and is comprised of cognitive, motor and behavioral symptoms. The milder form of neurocognitive impairment, minor cognitive motor disorder (MCMD), remains prevalent in the HAART era, affecting an estimated 40% - 50% of HIV-infected individuals, while the more severe forms of dementia have been substantially reduced [4]. The occurrence of MCMD, despite the efficacy of HAART therapy in controlling the viral load, suggests that the CNS viral load is not the only factor determining the prevalence of HAND. In fact, some studies suggest that glial activation shows better correlation with the severity of HAND than the amount of HIV replication in brain [5,6].

Astrocytes are the most abundant cell type in the brain and play a primary role in the maintenance of homeostasis in the brain. They regulate synaptic transmission, maintain the integrity of the blood–brain barrier (BBB) and protect neurons by clearing toxic compounds [7]. HIV has been shown to produce restricted infection of astrocytes that can become productive in a supportive environment [8,9]. Upon HIV entry into the CNS, microglial cells, perivascular macrophages and astrocytes become activated and release a myriad of neurotoxins such as quinolinic acid, TGF-beta, TNF-α, MCP-1, RANTES/CCL5, IL-8, IP-10 and NO [10-15]. The HIV-infected cells in the CNS also release viral particles such as gp120 and Tat in the brain microenvironment. These viral particles have been demonstrated to elicit inflammatory responses from the glial cells and have also been implicated in neuronal apoptosis [16,17]. Given the abundance and importance of astrocytes in the CNS, their dysregulation could have profound and lasting consequences; for this reason, these cells are widely believed to be a major cell type involved in the progression of HAND. In fact, previous work from our laboratory has demonstrated a role for HIV-1 gp120 in the production of IL-6, IL-8 and CCL5 in astrocytes [18-20].

Viral protein R (Vpr) is a 96-amino-acid protein that is highly conserved among lentiviruses. The role of Vpr in HIV infection and replication is multifaceted and includes such functions as cell cycle arrest at the G2 phase, transport of the pre-integration complex into the nucleus and transactivation of HIV-1 long-terminal repeat [21-23]. The importance of Vpr in HIV pathogenesis is underscored by the findings that HIV infection *in vitro* is enhanced by Vpr [24,25]. Vpr has been found in the different brain cell types including astrocytes of HAND patients [26]. Some pathological changes associated with Vpr in the brain include neuronal apoptosis, impaired axonal growth, elevation of intracellular calcium and increased production of reactive oxygen species in neuronal cells [27-29]. Furthermore, Vpr was recently shown to induce IL-6 in monocyte-derived macrophages (MDM), which can reactivate virus production from latently infected cells [30].

CCL5, also known as RANTES, is a multifunctional chemokine with evidence available for both harmful and beneficial actions in the CNS. A study by Si et al. provided indirect evidence for the potential of Vpr to induce RANTES/CCL5 in human microglial cells, where Vpr deleted HIV-1 showed much lower levels of CCL5 when compared with intact HIV-1 containing Vpr [31]. Though the roles of Tat and gp120 have been extensively studied, little work has been done on the role of Vpr on the astrocytes. Given the potential role of Vpr in the activation of astrocytes and microglial cells, it seems likely that Vpr may play a critical role in the development of HAND. In view of this, we sought to address the direct effect of Vpr overexpression on the induction of chemokine RANTES/CCL5 in astrocytes. In this report, we also examined several distinct signaling mechanisms that contributed to the induction of CCL5 in astrocytes.

## Materials and methods

### Cell culture and reagents

SVGA, a clone of the human fetal astrocytic cell line (SVG) [32], was kindly provided by Dr. Avindra Nath. These cells were maintained in Dulbecco’s modified Eagle medium (DMEM, Cellgro) containing 10% FBS, 1% L-glutamine, 1% non-essential amino acids, 1% sodium bicarbonate and gentamycin (50 μg/ml) in a humidified incubator at 37°C and 5% CO_2_. Lipofectamine™ 2000 was obtained from Invitrogen Inc. (Carlsbad, CA). Inhibitors for NF-κB (SC514: IKK-β), P38/MAPK (SB203580), PI3K (LY294002) and JNK (SP600125) were obtained from Cayman Chemicals (Ann Arbor, MI, USA). Pre-designed siRNAs for NF-κB (p50, p65), p38-MAPK (p38α, p38β, p38γ, p38δ), Akt (Akt-1, Akt-2, Akt-3) and AP-1 were purchased from Thermo Fisher Scientific Inc. (Waltham, MA). All the experimental protocols used in this study were approved by the Institutional Biosafety Committee (IBC) at UMKC.

### Construction of the HIV-1 Vpr plasmid

The Vpr expression plasmid was generated by amplification of the Vpr sequence from HIV-1 IIIB for cloning into the pcDNA3.1+ backbone. Briefly, H9/IIIB cells were cultured for RNA isolation. RNA was reverse transcribed and amplified by PCR using forward and reverse primers specific for the 5’ end (including the start codon) and 3’ end (including a stop codon) of the Vpr coding sequence, respectively. PCR product was verified by gel analysis and cloned directionally into pcDNA3.1D TOPO cloning vector (Invitrogen). Clones were sequenced to assess codon integrity. The pcDNA3.1/Vpr96 clone was prepared for transfection by the Endo Free Plasmid Mega kit (Qiagen) using the standard protocol to obtain a high yield of endotoxin free plasmid.

### Transfection

SVGA cells were transiently transfected with Lipofectamine™ 2000 as per the manufacturer’s protocol. Briefly, 0.8 × 10^6^ cells were incubated with 1 μg Vpr plasmid and 4 μl of lipofectamine in 1 ml serum-free medium for 5 h. The transfection was terminated by replacing the transfection medium with an equal volume of complete medium. The expression level of CCL5 was measured at 1, 3, 6, 12, 24, 48 and 72 h post transfection. For inhibition experiments, the cells were treated with 10 μM inhibitor (SB203580, LY294002, SP600125 and SC514) 1 h prior to the transfection with the plasmid encoding Vpr. For siRNA inhibition experiments, the cells were incubated for 24 h in transfection medium with 50 nmol of siRNA, followed by 24 h of incubation in complete medium before transfection with the plasmid encoding Vpr. For inhibitor and siRNA experiments, the mRNA expression level of CCL5 was determined at 6 h, while CCL5 protein levels were measured at 48 h post-transfection. Mock-transfected cells were used as a control to calculate the expression of CCL5.

### Real-time RT-PCR and CCL5 protein determination

Total RNA was extracted from the SVGA cells using RNeasy mini kits (QIAGEN, Valencia, CA) using the manufacturer’s protocol. Then 150 ng of RNA was amplified using Bio-Rad iCycler using the primer sequences that were commercially synthesized. The primer sequences for CCL5 and PCR conditions used have been previously described [19]. HPRT (hypoxanthine-guanine phosphoribosyl transferase) was used as a housekeeping control, and the 2^-∆∆Ct^ method was employed to calculate the expression levels of CCL5 [33]. The primer sequences and PCR conditions for p38 and Akt isoforms used in this study with slight modification have been previously described [34,35]. The annealing temperatures of the primers were selected based on their amplification in gradient PCR. The annealing temperatures in this study were 57.5°C (Akt-1, 2, 3 and p38δ), 58°C (p38α and p38γ) and 59.7°C (p38β) for 30 s.

The cell culture supernatants were centrifuged twice at 3,000 rpm and stored at -80°C. Centrifugation was done to remove the cell debris that has the potential to block the probe used in Bio-Plex system. The concentration of extracellularly secreted cytokines in the supernatants was measured by multiple cytokine assay kits (Bio-Rad, Hercules, CA) using the manufacturer’s protocol and previously published method [19]. The CCL5 concentration was determined based on 5PL standard curve using Bio-Plex manager 5.0 software.

### Agarose gel electrophoresis

The PCR products for p38 and Akt isoforms were electrophoresed on 1.5% agarose gel for 1 h at 100 V. The gel was stained with 0.005% ethidium bromide and was visualized using the Alpha Innotech FluorChem® imager for 8 ms under UV light.

### Immunocytochemistry

On glass cover slips, 0.6 × 10^6^ cells were cultured in a six-well plate and transfected with the plasmid encoding Vpr. After 5 h, the transfection medium was replaced with complete DMEM containing 1 mg/ml GolgiStop™ (BD Biosciences, San Jose, CA) to prevent the release of CCL5. After 6 h, the cells were fixed with 1:1 ice-cold methanol:acetone for 20 min at 20°C. The cells were then permeabilized with PBST (PBS containing 0.1% TritonX-100) and blocked with 1% BSA in PBST for 30 min at room temperature. The cells were washed 5× with PBS after fixation and permeabilization. The cells were incubated with a mixture of a rabbit polyclonal antibody to CCL5 (1:500; P230E, Thermo Scientific, Rockford IL USA) and mouse MAb anti-GFAP (1:1,000; ab106509, Abcam) overnight in a humidified chamber. The cells were washed 5× with PBST and incubated with an anti-rabbit antibody conjugated with Alexa Fluor 488 (1:1,000; Cell Signaling Technology) and an anti-mouse antibody conjugated with Alexa Fluor 555 (1:1,000; Cell Signaling Technology) for 1 h at room temperature in the dark. All the antibodies were diluted in 1% BSA containing PBS. The cells were washed 5× with PBST and mounted on a glass slide with 10 μl of Vectashield mounting medium containing DAPI (Vector Laboratories, Burlingame, CA). The images were captured using Leica TCS SP5 II (Leica Microsystems, Wetzler, Germany) on an inverted microscope platform with 40× zoom lens. The intensity of images was calculated using the Multi Measure tool from NIH ImageJ software. The values were normalized to GFAP.

### Western blotting

SVGA astrocytes were either mock transfected or were transfected with a plasmid encoding HIV-1 Vpr, and the cells were harvested after 6 h, followed by the separation of nuclear and cytosolic extracts using the NE-PER nuclear extraction kit (Pierce, Rockford, IL). Total cell extracts were prepared by adding RIPA buffer (Boston Bioproducts, Ashland, MA). The lysates were homogenized for 15 s and centrifuged at 14,000 × g for 15 min to eliminate cell debris. Protein concentrations were determined using the BCA protein assay kit (Pierce, Rockford, IL), and 20 μg of protein was resolved on 10% SDS-PAGE (75 V for 3 h). For immunoblotting, proteins were transferred (350 mA for 1.5 h) onto a PVDF membrane and blocked overnight at 4°C with 5% nonfat milk in PBST (PBS with 0.075% Tween 20). The membrane was incubated with primary antibodies against p50 (Santa Cruz Biotechnology Inc., 1:1,500) and c-fos (Cell Signaling Technology, 1:1,500) for 3 h at room temperature and washed 5× with PBST, followed by 2 h incubation in an appropriate concentration of secondary antibodies conjugated to horseradish peroxide. The membrane was then washed 5× with PBST, and the bands were visualized using the BM chemiluminescence Western blotting substrate (POD, Roche Applied Sciences; Indianapolis, IN). FluorChem HD2 software (Alpha Innotech, San Leandro, CA) was used for the quantification of the bands.

### Statistical analysis

The results are expressed as mean ± SE for three independent experiments with each experiment done in triplicate. The statistical significance was calculated using Student’s *t*-test, and a *p* value ≤ 0.05 was considered significant.

## Results

### Time-dependent induction of CCL5 by HIV-1 Vpr in SVGA astrocytes

**Figure 1:**
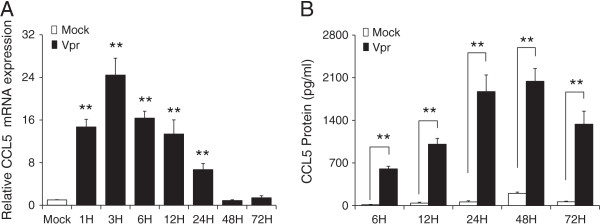
**Time-dependent induction of CCL5 by HIV-1 Vpr in SVGA astrocytes.** SVGA cells were mock transfected or transfected with a plasmid encoding HIV-1 Vpr. Cells were harvested at 1, 3, 6, 12, 24, 48 and 72 h post-transfection, and CCL5 expression levels were determined using real-time RT-PCR. The supernatants were also collected at 6, 12, 24, 48 and 72 h post-transfection, and protein concentration of CCL5 was determined using Bioplex assay. **(A)** mRNA expression levels calculated relative to mock-transfected controls; **(B)** protein concentration for CCL5. The *bars* represent the mean ± SE of three independent experiments done in triplicate. Statistical significance was determined using Student’s *t*-test, **p < 0.01, *p < 0.05.

The expression levels of CCL5 are known to be increased in the CSF of individuals suffering from HAND. We transfected SVGA astrocytes with a plasmid encoding HIV-1 Vpr using Lipofectamine 2000 reagent to ascertain whether viral protein R has any role in increased CCL5 expression. The transfection efficiency as determined by GFP transfection followed by BD FACScanto flow cytometric analysis was in the range of 60-80% (data not shown). CCL5 mRNA expression was determined at 1, 3, 6, 12, 24, 48 and 72 h post-transfection (Figure 1A). The CCL5 mRNA expression level peaked at 3 (24.43 ± 3.18-fold) and declined thereafter to reach the basal level at 48 h. The increase in RNA level was further confirmed by determining the protein concentrations of CCL5 in cell culture supernatants. The supernatants were collected and analyzed at 6, 12, 24, 48 and 72 h after Vpr transfection of SVGA astrocytes (Figure 1B). We observed significantly higher levels of CCL5 in Vpr-transfected astrocytes compared to mock transfected at time as low as 6 h. The CCL5 protein concentration was higher at all time intervals analyzed, and the peak CCL5 concentration was seen at 48 h post transfection (2,040.5 ± 209.6 pg/ml) compared to mock-transfected controls (200.7 ± 20.1 pg/ml).

### Immunocytochemistry for HIV-1 Vpr-mediated induction of CCL5 in SVGA astrocytes

**Figure 2:**
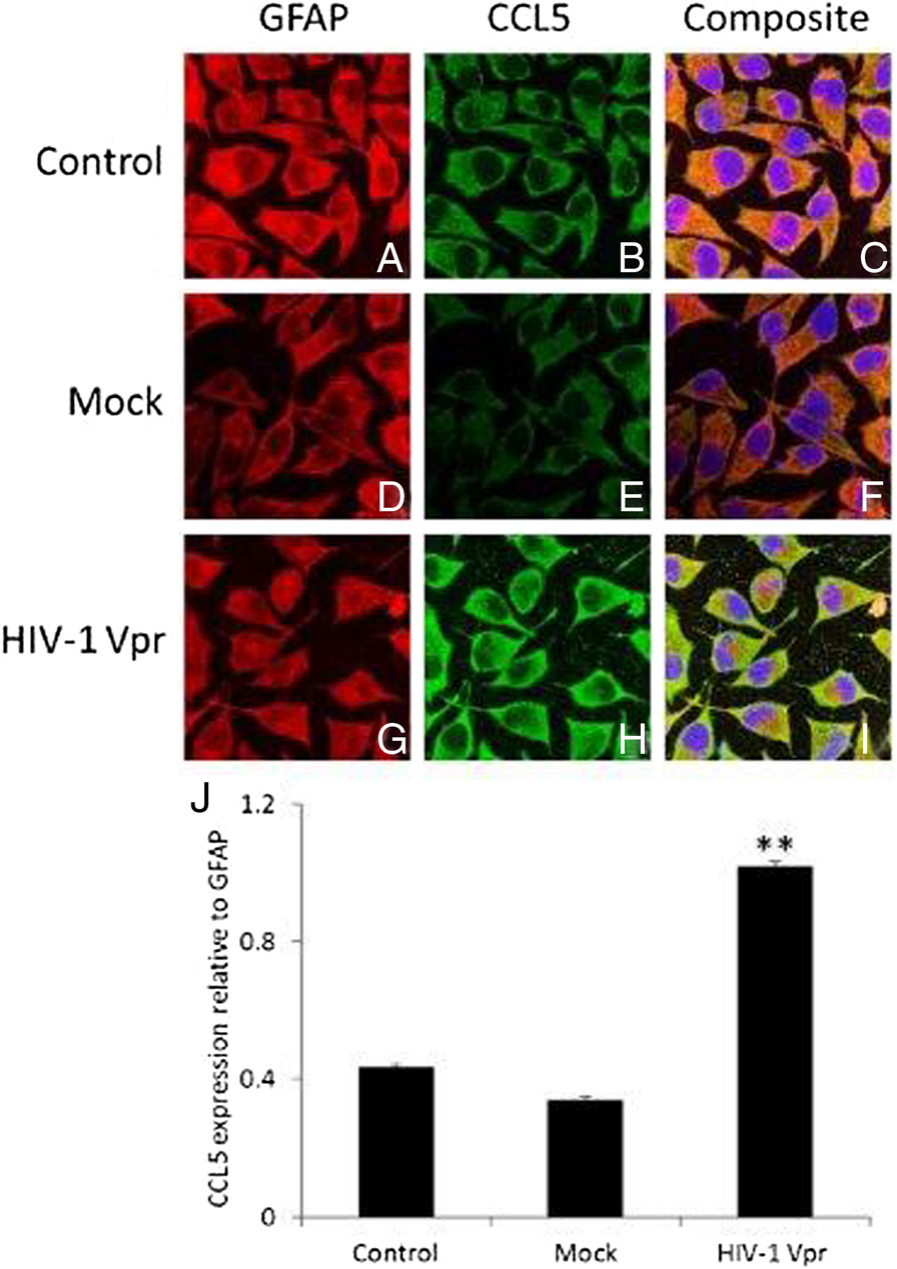
**Immunocytochemistry for HIV-1 Vpr-mediated induction of CCL5 in astrocytes.** SVGA astrocytes were cultured on cover slips and were either mock transfected **(D-F)** or were transfected with a plasmid encoding Vpr **(G-I)**. Non-transfected cells were used as control **(A-C)**. The cells were stained for nucleus (*blue*); CCL5 (*green*) and GFAP (*red*), and the images were captured using a Leica TCS SP5 II on an inverted microscope platform with a 40× zoom oil emersion lens. The Image J software was used to get the merged images, and the Multi Measure tool in Image J was used to quantify the intensities. **(J)** The image intensities are plotted relative to GFAP. The *bars* represent the mean ± SE of three independent images. Statistical significance was determined using Student’s *t*-test, **p < 0.01.

In order to further confirm HIV-1 Vpr-mediated increased expressions of CCL5, we performed immunocytochemistry on SVGA astrocytes after transfection with a plasmid encoding Vpr. The cells were immunostained with a cocktail of GFAP- and CCL5-specific antibodies. These proteins were visualized by staining with secondary antibodies conjugated to Alexa Fluor 488 (*green*) and Alexa Fluor 555 (*red*) for CCL5 and GFAP, respectively. DAPI staining was used to visualize the nuclei of the cells. A representative staining is shown in Figure 2. A strong yellow signal in the merged images signifying the accumulation of CCL5 that was co-localized with GFAP was seen in the astrocytes transfected with Vpr (Figure 2G-I) as compared to mock-transfected (Figure 2D-F) or un-transfected controls (Figure 2A-C). Our results also indicated that mock transfection caused a slight but statistically non-significant decrease in CCL5 expression (Figure 2J). On the other hand, relative CCL5 expression in HIV-1 Vpr-transfected cells was 2.4- and 3.1-fold higher compared to control and mock-transfected cells, respectively (Figure 2J).

### HIV-1 Vpr-mediated upregulation of CCL5 was abrogated with inhibitor and siRNA against the NF-κB pathway

**Figure 3:**
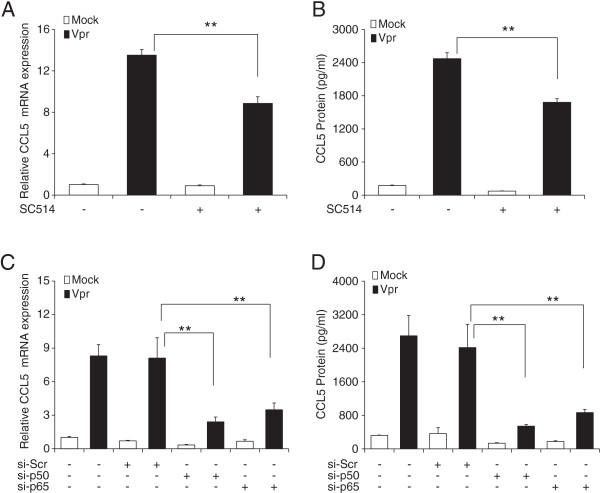
**Involvement of NF-κB in HIV-1 Vpr-mediated upregulation of CCL5 in astrocytes.** SVGA astrocytes were pre-treated with the chemical inhibitors or transfected with siRNA targeting the NF-κB pathway, followed by mock transfection or transfection with a plasmid encoding Vpr. **(A)** and **(B)** Effect of chemical inhibitor of the NF-κB pathway, SC514 on mRNA expression and protein concentration for CCL5, respectively. **(C)** and **(D)** Effect of siRNA against p50 and p65 subunits of NF-κB on CCL5 mRNA expression and protein concentration, respectively. The *bars* represent the mean ± SE of three independent experiments done in triplicate. Statistical significance was determined using Student’s *t*-test, **p < 0.01, *p < 0.05.

To identify the role of NF-κB in HIV-1 Vpr-mediated upregulation of CCL5 in astrocytes, we tested SC514, which is a specific inhibitor of NF-κB activation. The concentration of inhibitor used was determined based on IC50 values and its effect on cell viability (data not shown). The cells were pre-treated 1 h with 10 μM of SC514 before Vpr transfection, and the inhibitor was present throughout the experiment. The CCL5 mRNA expression and protein concentration were measured at 6 and 48 h post transfection, respectively (Figure 3A, B). SC514 treatment significantly inhibited the production of CCL5 at mRNA (8.85 ± 0.64-fold) and protein level (1,681.9 ± 65.6 pg/ml) as compared to untreated controls (13.15 ± 0.54-fold, 2,471.6 ± 110.9 pg/ml). To further confirm the role of NF-κB, we transfected the cells with siRNA against p50 and p65 subunits of NF-κB for 48 h before transfecting them with a plasmid encoding Vpr (Figure 3C, D). HIV-1 Vpr caused reduced CCL5 mRNA expression in both p50 siRNA (2.4 ± 0.43-fold) and p65 siRNA (3.49 ± 0.61-fold) transfected cells as compared to those cells transfected with scrambled siRNA (8.11 ± 1.82-fold). We observed similar trend in the CCL5 protein levels as well with p50 siRNA (540.42 ± 41.39 pg/ml) and p65 siRNA (864.32 ± 77.43 pg/ml) showing statistically significant reductions as compared to scrambled siRNA transfected control (2,417.36 ± 553.39 pg/ml).

### Involvement of the p38-MAPK and AP-1 pathway in HIV-1 Vpr-mediated induction of CCL5 in astrocytes

**Figure 4:**
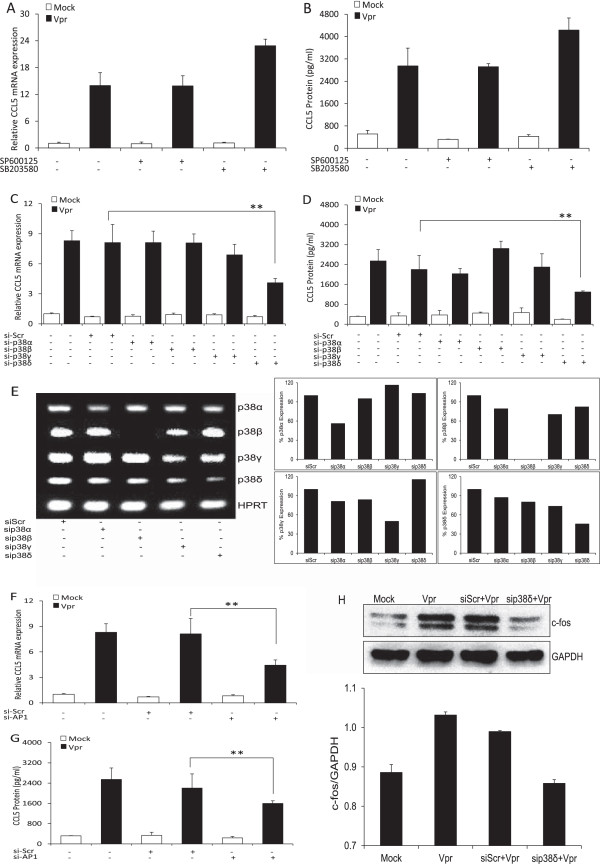
**Role of p38-MAPK and AP-1 in HIV-1 Vpr-mediated induction of CCL5 in astrocytes.** SVGA astrocytes were pre-treated with the chemical inhibitors for the JNK-MAPK (SP600125) and p38-MAPK (SB203580) pathway 1 h before mock transfection or transfection with a plasmid encoding HIV-1 Vpr. **(A)** and **(B)** Effect of chemical inhibitors on mRNA expression and protein concentration for CCL5, respectively. SVGA cells were also transfected with siRNA targeting p38 isoforms (α, β, γ, δ) and AP-1 followed by mock transfection or transfection with a plasmid encoding Vpr. **(C)** and **(F)** Effect of siRNAs on CCL5 mRNA expression; **(D)** and **(G)** effect on CCL5 protein concentration, respectively. **(E)** Specificity of p38 isoform siRNAs against respective subunits; **(H)** inhibition of AP-1 (c-fos) with the siRNA directed toward the p38δ subunit of p38-MAPK. The *bars* represent the mean ± SE of three independent experiments done in triplicate. Statistical significance was determined using Student’s *t*-test, **p < 0.01, *p < 0.05.

To dissect the upstream pathway involved in the production of CCL5 after Vpr transfection of SVGA astrocytes, we tested the chemical inhibitors for the MAPK (SP600125-JNK, SB203580-p38) pathway. The optimum concentration of inhibitor was determined based on cell viability and dose–response studies (data not shown). The cells were pre-treated for 1 h with 10 μM of inhibitors and then were either mock transfected or transfected with a plasmid encoding Vpr. No significant reductions (Figure 4A, B) were seen with either SP600125 (13.89 ± 0.85-fold, 2,919.9 ± 111.5 pg/ml) or SB203580 (22.89 ± 2.09-fold, 4,233.6 ± 433.3 pg/ml) as compared to untreated controls (13.99 ± 1.65-fold, 2,946.92 ± 639.9 pg/ml). For confirmation, SVGA cells were transfected with siRNA against p38-MAPK (α, β, γ and δ) isoforms (Figure 4C, D). Surprisingly, siRNA against the p38δ isoform showed inhibition at both the mRNA (4.11 ± 0.42-fold) and protein (1,298.2 ± 40.96 pg/ml) levels, which was not seen with chemical inhibitor against the p38 pathway. This was in confirmation of a previous report that SB203580 inhibits only the α and β but not the γ and δ isoforms of the p38 pathway [36]. In order to determine the specific silencing effect of individual p38 isoform-specific siRNA, we amplified the RNA from the cells depleted with different p38 isoforms. The knockdown of the target was assessed by resolving the product on agarose gel (Figure 4E) with HPRT as a housekeeping control. To ascertain the downstream signaling molecule of p38δ MAPK, we transfected the cells with siRNA against AP-1 transcription factor and determined the effect of Vpr transfection at 6 h for mRNA and 48 h for protein expression (Figure 4F, G). Statistically significant reduction was seen with siRNA directed against AP-1 at mRNA (4.43 ± 0.62-fold) and protein (1,598.34 ± 104.9 pg/ml) levels. This was further confirmed by determining the levels of c-fos, one of the major subunits of AP-1, after the transfection with p38δ-specific siRNA (Figure 4H). The transfection of HIV-1 Vpr in astrocytes led to an increase in the levels of c-fos, which was partially reversed by p38δ-specific siRNA but not with scrambled siRNA.

### PI3K/Akt-mediated activation of NF-κB plays a role in the HIV-1 Vpr-mediated expression of CCL5 in SVGA astrocytes

**Figure 5:**
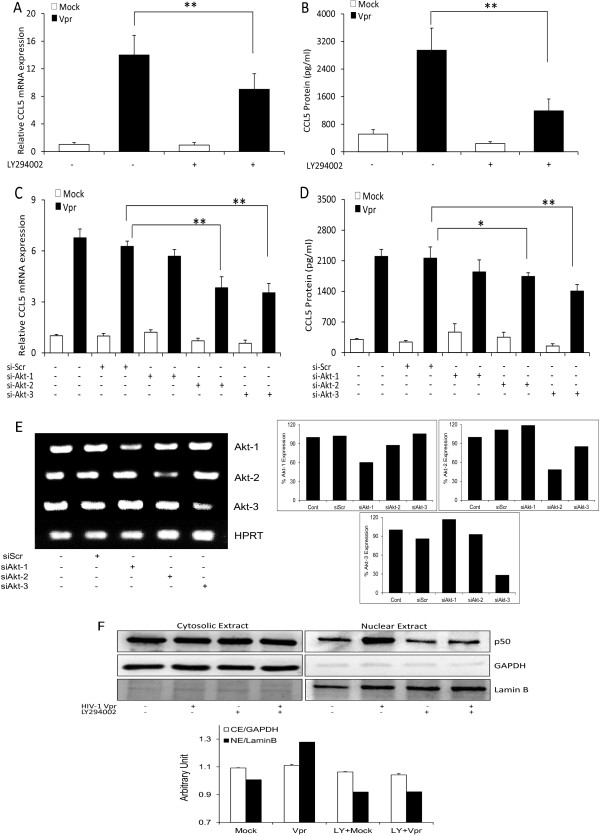
**Activation of NF-κB involves PI3K/Akt signaling in HIV-1 Vpr-mediated induction of CCL5 in astrocytes.** SVGA astrocytes were pre-treated with the chemical inhibitor for the PI3K/Akt pathway (LY294002) 1 h before mock transfection or transfection with a plasmid encoding HIV-1 Vpr. **(A)** and **(B)** Effect of LY294002 on mRNA expression and protein concentration for CCL5, respectively. SVGA cells were also transfected with siRNA targeting Akt isoforms (Akt-1, Akt-2 and Akt-3) followed by mock transfection or transfection with a plasmid encoding Vpr. **(C)** and **(D)** Effect of siRNAs on CCL5 mRNA expression and protein concentration, respectively. **(E)** Specificity of siRNA for Akt isoforms against individual subunits. **(F)** Inhibition of NF-κB (p50) nuclear translocation with LY294002. The *bars* represent the mean ± SE of three independent experiments done in triplicate. Statistical significance was determined using Student’s *t*-test, **p < 0.01, *p < 0.05

To understand the upstream signaling responsible for the activation of NF-κB in HIV-1 Vpr-mediated induction of CCL5 in astrocytes, we pre-treated the cells with the PI3K/Akt inhibitor LY294002 (10 μM). The optimal dose concentration was determined based on the dose–response and cell viability studies. Pre-treatment with LY294002 (Figure 5A, B) substantially reduced the expression of CCL5 (9.02 ± 1.30-fold, 1,188.8 ± 343.1 pg/ml) as compared to untreated controls (13.99 ± 1.65-fold, 2,946.92 ± 639.9 pg/ml) after transfection of astrocytes with HIV-1 Vpr. Further, to corroborate the inhibition seen with LY294002, we transfected Vpr into the cells that were already transfected with siRNA against Akt isoforms (Akt-1, Akt-2 and Akt-3) or scrambled control (Figure 5C, D). The astrocytes transfected with siRNAs that targeted Akt-2 (3.83 ± 0.64-fold; 1,742.8 ± 82.6 pg/ml) and Akt-3 (3.54 ± 0.53-fold; 1,408.2 ± 150.8 pg/ml) showed significant abrogation of CCL5 at both the mRNA and protein levels as compared to scrambled siRNA-transfected control (6.26 ± 0.31-fold, 2,158.9 ± 257.5 pg/ml) after Vpr transfection. The slight inhibition seen with Akt-1 isoform (5.69 ± 0.38-fold; 1,846.3 ± 274.1 pg/ml) was not statistically significant. Specificity of the siRNAs for Akt isoforms was determined by amplifying the silenced RNA using Akt isoform-specific primers. The products were then resolved on the agarose gel, stained with ethidium bromide and visualized under UV light (Figure 5E). Amplification of HPRT was used as a housekeeping control.

**Figure 6:**
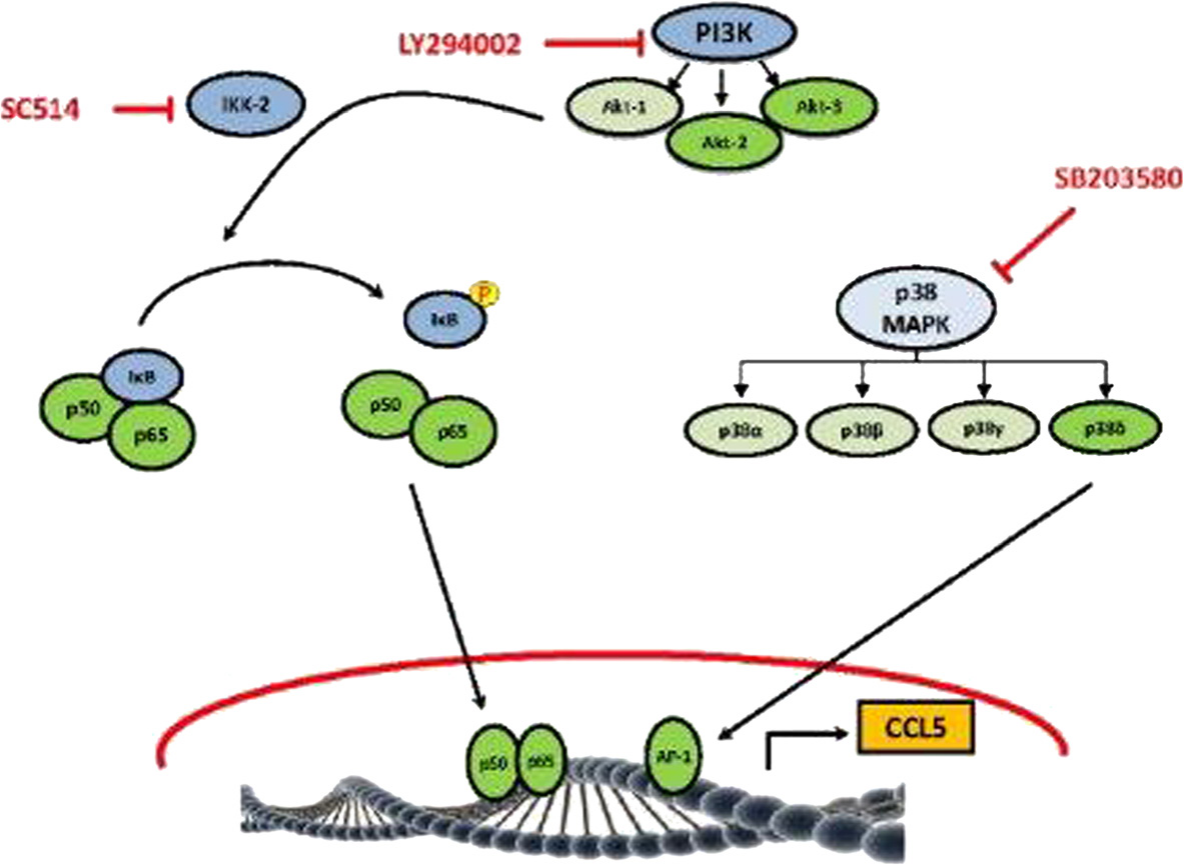
**Schematic of the signaling pathways involved in the induction of CCL5 by HIV-1 Vpr in astrocytes.** HIV-1 Vpr activates p38-MAPK- and PI3K/Akt-related signaling pathways in SVGA astrocytes leading to the activation of transcription factors AP-1 and NF-κB, respectively. NF-κB and AP-1 bind to the promoter region of CCL5 and increase its production in astrocytes. Blue color represents the use of chemical inhibitor, while green color represents the use of siRNA. Involvement of particular signaling is shown in darker shades, while lighter shades imply no involvement.

To confirm whether PI3K/Akt signaling is involved in the activation NF-κB, LY294002-treated cells were analyzed for the translocation of p50 from cytosol to nucleus. PI3K/Akt inhibition by LY294002 substantially reduced p50 translocation as compared to inhibitor-untreated controls (Figure 5F). This clearly demonstrates that NF-κB activation in response to HIV-1 Vpr was mediated though PI3K/Akt signaling. The schematic representation (Figure 6) depicts that HIV-1 Vpr induces the expression of CCL5 in astrocytes through the activation of transcription factors NF-κB and AP-1 through the involvement of signaling intermediates PI3K/Akt and p38δ, respectively.

## Discussion

The primary goal of this study was to determine the effect of HIV-1 Vpr on the CCL5 expression in astrocytes and to unravel the underlying mechanism(s). In addition to CCL5, we also analyzed the expressions of TNF-α and IL-1β in the astrocytes with HIV-1 Vpr in real-time RT-PCR and multiple cytokine assay. However, these cytokines were generally found to be unaffected by HIV-1 Vpr and thus were not considered for further studies. HIV-1 Vpr is known to be apoptogenic and has been implicated in apoptosis of a variety of cell types including T-cells, neurons and astrocytes [37-39]. Vpr is shown to play a role in neuroinflammation associated with HIV-1 by inducing the production of many neurotoxins such as IL-6, IL-8 and ROS [30,40]. The importance of Vpr in CCL5 induction is highlighted by the fact that Vpr-deleted HIV-1 produces much lower amounts CCL5 as compared to the intact HIV-1 in microglial cells [31]. Our results have unequivocally shown that HIV-1 Vpr induces the production of CCL5 in astrocytes in a time-dependent manner at both RNA and protein levels. CCL5 is widely established as a proinflammatory chemokine and has been shown to be involved in neuroinflammatory conditions. However, in the context of HIV-1 infection, its role has been contradictory. CCL5 at higher concentrations ranging from 500 ng/ml to 5 μg/ml increases the permissibility of cells to HIV infection [41]. On the other hand, CCL5 at 200 ng/ml – 500 ng/ml is shown to protect the neuronal cells from HIV-1 gp120-mediated cell death [42,43]. Also CCL5 treatment of the differentiated neuronal cell line at 300 ng/ml has been shown to upregulate genes involved in neuronal survival and neurite outgrowth [44]. The CCL5 concentration found in many neuropathological settings and considered as neurotoxic is below 50 ng/ml. In fact, it has been shown that 50 ng/ml CCL5 induces apoptosis in neuroblastoma cells [45]. We found a CCL5 concentration of 2–3 ng/ml at peak induction after introduction of Vpr in astrocytes, which might be closely related to neuroinflammatory as opposed to the neuroprotective scenario. In T-cells, CCL5 has been shown to be present in unique storage vesicles and rapidly released after T-cell activation [46]. However, in our experimental conditions, CCL5 was observed to be secretory soon after production as we detected significant concentration of CCL5 within 6 h. Therefore, It seems that the CCL5 storage phenomenon in distinct secretory compartments may be cell-type specific.

NF-κB is a ubiquitous transcription factor, involved in the regulation of myriad of inflammatory genes as well as cell survival signaling pathways. It plays an essential role in the expression of the CCL5 gene as well [47]. To explore the possibility of involvement of NF-κB in CCL5 induction by HIV-1 Vpr in astrocytes, we employed SC514, which is a specific inhibitor for the IKK-2 pathway of NF-κB activation [48]. IKKs are upstream kinases responsible for phosphorylation and proteasomal degradation of IκB-α and subsequent activation of NF-κB. NF-κB complex consists of p50 and p65 subunits attached to inhibitory IκB-α, which retains them in the cytosol. This complex gets activated by the removal of IκB-α, translocates to the nucleus and binds to the promoter regions of specific genes [49]. The reduction in CCL5 expression by SC514 therefore confirms the involvement of the NF-κB pathway in HIV-1 Vpr-mediated production of CCL5 in astrocytes. Our results using p50- and p65-specific siRNA also demonstrate the direct involvement of NF-κB in CCL5 expression.

Recently, it has been reported that CCL5 expression in astrocytes can be blocked by the inhibitors of the MAPK and PI3K pathway [50]. The CCL5 promoter contains binding sites not only for NF-κB, but also for CREB (cAMP response element-binding protein), AP-1 (activator protein 1), C/EBP (CCAAT/enhancer binding protein) and IRF (interferon regulatory factor) [51]. These transcription factors are known to involve upstream signaling through the MAPK and PI3K/Akt pathway. In this study, the treatment of astrocytes with LY294002 (PI3K/Akt) but not with SB203580 (p38 MAPK) and SP600125 (JNK MAPK) inhibited the CCL5 expression in response to HIV-1 Vpr. These results clearly suggest that PI3K/Akt but not JNK/MAPK is involved in NF-κB activation in our system. In our attempt to further dissect the involvement of PI3K/Akt, we employed Akt-specific siRNAs. Akt, also known as protein kinase B, is a family of serine/threonine kinases comprising three isoforms, Akt-1, Akt-2 and Akt-3. They differ from each other in only one amino acid residue in their phosphorylation/activation site, Akt-1 (ser-473, thr-308), Akt-2 (ser-474, thr-309) and Akt-3 (ser-472, thr-305) [52]. They also differ in their subcellular localization in a tissue-specific manner, with Akt-3 being the most abundant isoform in the brain [52,53]. It has been shown that Akt-3-deficient mice have smaller brains with suppressed inflammatory responses in experimental autoimmune encephalomyelitis [53,54]. Recently, Akt-2 deficient macrophages have been shown to be hyporesponsive to LPS and produce lower levels of IL-6 and TNF-α [53]. In our study, siRNA mediated knockdown of Akt-2 and Akt-3 isoforms but not Akt-1 showed suppression of CCL5, which is in consistent with earlier reports that Akt-2 and Akt-3 play an important role in regulation of cytokine gene expression [54,55].

Our results showing only partial abrogation of CCL5 expression by SC514, LY294002, sip50 and sip65 suggest the possibility that other signaling mechanisms are also involved in HIV-1 Vpr-mediated CCL5 upregulation. Therefore, we explored various p38 MAP kinases. There are four isoforms of the p38-MAPK pathway, p38α, p38β, p38γ and p38δ, which can be activated by stress and are distributed in a tissue-specific manner. SB203580 (a p38 chemical inhibitor) did not show any CCL5 inhibition, but it is a known inhibitor of only p38α and p38β isoforms with no or minimal inhibition at higher concentrations on p38γ and p38δ isoforms [36]. We therefore used siRNAs against each p38 isoform. Our results with p38δ siRNA raised the possibility of involvement of another transcription factor (AP-1) because the CCL5 promoter contains an AP-1 responsive element and has been shown to be involved in the production of CCL5 [47]. This was confirmed by siRNA-mediated AP-1 knockdown. The p38δ and AP-1 connection has been shown in other systems as well, as it has been shown to regulate keratinocyte differentiation through the AP-1 transcription factor [56]. Furthermore, synthetic Vpr protein has been shown to activate AP-1, which in turn stimulates HIV-1 transcription in monocytes and macrophages [57]. We also found the reduction in the expression of c-fos subunit of AP-1 with the siRNA directed against p38δ. This clearly demonstrates the involvement of AP-1 in HIV-1 Vpr-mediated induction of CCL5 in astrocytes. Further, the activation and nuclear translocation of the p50 subunit of NF-κB involved PI3K/Akt signaling were illustrated with the reduction of p50 nuclear levels in the presence of LY294002. This provides direct evidence for the involvement of PI3K/Akt in the activation of NF-κB with the transfection of astrocytes with HIV-1 Vpr. Our studies are in accordance with the previous report suggesting the involvement of HIV-1 Vpr in the activation of transcription factors such as NF-κB and AP-1 in primary macrophages [57].

## Conclusions

In summary, we have shown that HIV-1 Vpr induces CCL5 expression in astrocytes in a time-dependent manner. Furthermore, CCL5 expression involved the transcription factors NF-κB and AP-1. AP-1 was shown to be activated by p38δ, while NF-κB activation involved signaling through the PI3K/Akt pathway (Figure 6). These studies are important for the development of adjunct therapy as we have identified different steps that could be targeted to suppress CCL5 expression.

## Competing interest

The authors declare that they have no competing interests.

## Authors’ contributions

MG executed most of the experiments and analyzed the data shown in this manuscript. He also wrote the first draft of the manuscript. RN constructed HIV-1 Vpr plasmid. AS helped MG to learn some of the techniques used for this project. VRA participated in the project design. AK conceptualized the project and finalized the manuscript. All the authors have read and approved the final version of the manuscript.
